# Veränderungen des Immunsystems im Alter: Immunseneszenz und Auswirkungen auf Impfungen

**DOI:** 10.1007/s00391-025-02496-4

**Published:** 2025-09-05

**Authors:** Birgit Weinberger, Peter Dovjak

**Affiliations:** 1 Institut für Biomedizinische Alternsforschung, Universität Innsbruck, Rennweg 10, 6020 Innsbruck, Österreich; 2 Salzkammergut Klinikum Gmunden, Gmunden, Österreich

**Keywords:** Hämatopoese, Autoimmunität, Impfstoffe, Inflammaging, Impfantwort, Hematopoiesis, Autoimmunity, Vaccines, Inflammaging, Vaccination response

## Abstract

Im Alter kommt es zu komplexen Veränderungen des angeborenen und adaptiven Immunsystems. Das trägt dazu bei, dass Infektionen bei älteren Menschen häufiger auftreten und schwerer verlaufen. Gleichzeitig treten chronische, subklinische Entzündungen auf, die altersassoziierte Erkrankungen begünstigen. Altersassoziierte Veränderungen des Immunsystems beeinflussen auch die Entstehung, das Wachstum und die Metastasierung von malignen Tumoren. Darüber hinaus gibt es Hinweise, dass die Alterung des Immunsystems zur Entwicklung von Autoimmunität beiträgt. Impfungen sind eine wichtige Präventionsmaßnahme zum Erhalt der Gesundheit und zur Verbesserung der Lebensqualität, nicht nur, aber v. a. im Alter. Die Entwicklung optimierter und neuer Impfstoffe für die ältere Bevölkerung hat in den letzten Jahren und Jahrzehnten große Fortschritte gebracht. Die Durchimpfungsraten bei älteren Erwachsenen liegen jedoch für alle Impfungen deutlich unter den angestrebten Zielen.

## Abstract (en)

Complex changes of the innate and adaptive immune system occur in old age and contribute to the increased frequency and severity of infections in older adults. At the same time, chronic, subclinical inflammation occurs, which promotes age-related diseases. Age-associated changes in the immune system also influence the formation, growth and metastasis of malignant tumors. Furthermore, there are indications that the aging of the immune system contributes to the development of autoimmunity. Vaccinations are an important preventive measure for maintaining health and improving the quality of life, not only but especially in old age. The development of optimized and new vaccines for the older population has made great progress in recent years and decades; however, vaccination rates among older adults are well below target for all vaccinations.

## Lernziele

Nach Lektüre dieses Beitrags ...

- verstehen Sie den Ablauf einer Immunreaktion.
- können Sie die wichtigsten altersassoziierten Veränderungen des Immunsystems benennen.
- verstehen Sie, wie Impfungen vor Erkrankungen schützen.
- kennen Sie einige Impfstoffe, die speziell für ältere Erwachsene entwickelt wurden.

## Einleitung

Mit zunehmendem Alter verändert sich das Immunsystem, was dazu beiträgt, dass **Infektionen**

Infektionen

bei älteren Menschen häufiger auftreten und oft schwerer verlaufen. Infektionen bergen außerdem zusätzliche Risiken, die über die akute Erkrankung hinausgehen. Auch die Kontrolle von Tumorzellen ist beeinträchtigt, und altersbedingte immunologische Veränderungen können **Autoimmunerkrankungen**

Autoimmunerkrankungen

fördern. Gleichzeitig treten chronische, subklinische Entzündungen auf, die altersassoziierte Erkrankungen begünstigen.

Ein besseres Verständnis der Immunseneszenz kann dazu beitragen, die Lebensqualität im Alter zu verbessern und Erkrankungen vorzubeugen. Dieser Beitrag beleuchtet die wichtigsten Veränderungen des Immunsystems im Alter, deren Folgen und den Stellenwert von Impfungen für ältere Erwachsene.

## Veränderung des Immunsystems im Alter

Das **angeborene Immunsystem**

angeborene Immunsystem

ist die erste Verteidigungslinie, die „unspezifisch“ auf Pathogene reagiert. Es erkennt Strukturen (z. B. Zucker, Bestandteile der bakteriellen Zellwand etc.), die bei verschiedenen Pathogenen konserviert sind, und reagiert unmittelbar. Das **adaptive Immunsystem**

adaptive Immunsystem

benötigt mehr Zeit, entwickelt dann jedoch eine gezielte, spezifische Immunantwort. Die T‑ und B‑Zellen des adaptiven Immunsystems erzeugen durch somatische Rekombination eine große Vielfalt von Rezeptoren, die spezifische Antigene erkennen. Im Anschluss werden T‑ und B‑Zellen auf ihre Funktionalität getestet und potenziell autoreaktive Immunzellen eliminiert. Das **immunologische Gedächtnis**

immunologische Gedächtnis

ermöglicht bei wiederholtem Kontakt mit Erregern eine schnelle und effektivere Abwehr, was die Grundlage für Impfungen bildet.

Mit zunehmendem Alter kommt es zu komplexen Veränderungen des angeborenen und adaptiven Immunsystems, die als Immunseneszenz bekannt sind. Diese betreffen die Struktur und Funktionalität lymphatischer Organe, die Produktion von Immunzellen, löslichen Faktoren und Zytokinen, die Zusammensetzung der Zellpopulationen und die Funktionen einzelner Zellen [1].

### Hämatopoese

Alle Blutzellen entstehen im Knochenmark aus multipotenten **hämatopoetischen Stammzellen**

hämatopoetischen Stammzellen

(HSZ), die sich entweder selbst erneuern und wieder multipotente Stammzellen (Selbsterneuerung) hervorbringen oder zu myeloischen bzw. lymphoiden Vorläuferzellen differenzieren. **Myeloide Vorläuferzellen**

Myeloide Vorläuferzellen

entwickeln sich zu Erythrozyten, Megakaryozyten, aus denen Thrombozyten gebildet werden, Mastzellen, dendritischen Zellen (DZ) und Monozyten/Makrophagen. Aus den **lymphoiden Vorläuferzellen**

lymphoiden Vorläuferzellen

entstehen **natürliche Killerzellen**

natürliche Killerzellen

(NK-Zellen), T‑Zellen und B‑Zellen. Die B‑Zellen entwickeln sich im Knochenmark und gelangen über den Blutkreislauf in die Lymphorgane. Die T‑Zellen verlassen das Knochenmark in unreifem Zustand und reifen im Thymus, bevor sie in die Blutzirkulation und andere Lymphorgane entlassen werden. Mit zunehmendem Alter kommt es zu funktionellen Veränderungen der HSZ. Die Zahl der HSZ nimmt zu, während das Potenzial zur Selbsterneuerung abnimmt. Es werden bevorzugt myeloide Vorläuferzellen gebildet, was zu einer reduzierten Produktion von lymphoiden Zellen führt [2]. Die oben erwähnten Prozesse der somatischen Rekombination und Selektion finden für T‑Zellen in einem spezialisierten Organ, dem **Thymus**

Thymus,

statt. Dieser wird mit zunehmendem Alter zurückgebildet (Involution), was eine der auffälligsten, altersassoziierten Veränderungen des Immunsystems darstellt. Die Produktion neuer, naiver T‑Zellen wird weiter einschränkt, was mit einer geringeren Vielfalt des T‑Zell-Repertoires und weniger Flexibilität bei der **Antigenerkennung**

**Abb. 1:**
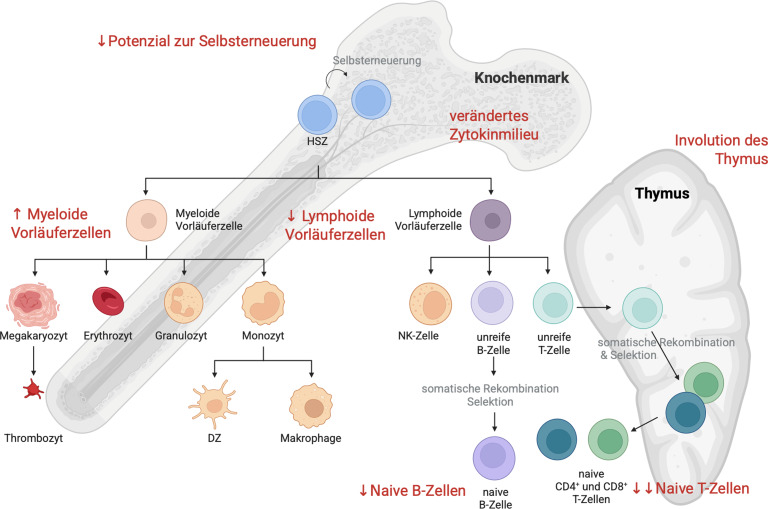
Schematische Darstellung der Hämatopoese im Knochenmark und der T‑Zell-Reifung im Thymus. Altersassoziierte Veränderungen sind in *roter Schrift* angeführt. *HSZ* hämatopoetische Stammzelle, *DZ* dendritische Zelle, *NK-Zelle* natürliche Killerzelle. Created in BioRender. Weinberger, B. (2025) https://BioRender.com/6xp37mg

Antigenerkennung

einhergeht [3]. Aufgrund einer veränderten Zytokinproduktion kommt es außerdem zur Selektion bestimmter HSZ-Klone mit genetischen und epigenetischen Veränderungen, was das Risiko für hämatologische Erkrankungen erhöht (Abb. 1).

### Immunreaktion am Beispiel der Immunantwort nach Impfung

Bei Kontakt mit einem Antigen kommt es zu Reaktionen des angeborenen und des adaptiven Immunsystems. Dieser Prozess soll am Beispiel einer Immunantwort nach Impfung mit einem Proteinantigen (z. B. Influenza) aufgezeigt werden, wobei auch die altersassoziierten Veränderungen der einzelnen Komponenten des Immunsystems beleuchtet werden.

Zellen des angeborenen Immunsystems (Granulozyten, Makrophagen und DZ) phagozytieren am Ort der Injektion die Impfstoffantigene. Sie produzieren **Zytokine**

Zytokine

und **Chemokine**

**Abb. 2:**
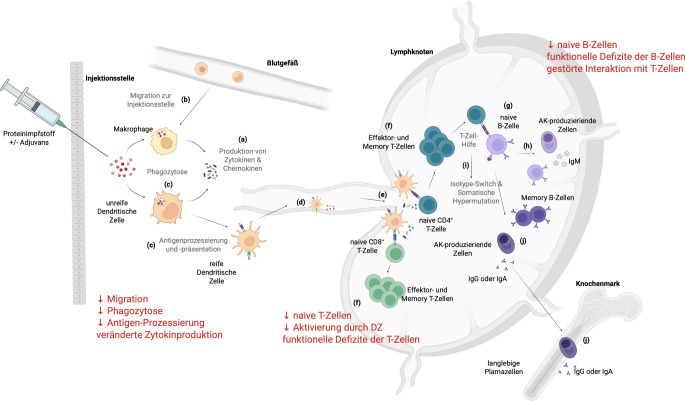
Schematische Darstellung der Immunantwort nach Impfung mit einem Proteinimpfstoff. Die einzelnen Schritte sind mit *(a)–(j)* bezeichnet und sind auch im Text so dargestellt. Altersassoziierte Veränderung sind in *roter Schrift* angeführt. *AK* Antikörper, *DZ* dendritische Zelle, *Ig* Immunglobulin. Created in BioRender. Weinberger, B. (2025) https://BioRender.com/j0zdalw

Chemokine

 (Abb. 2*(a)*), was eine lokale Entzündungsreaktion auslöst und weitere Immunzellen aus dem Blutkreislauf zur Injektionsstelle rekrutiert (Abb. 2*(b)*). Antigenpräsentierende Zellen, insbesondere **dendritische Zellen**

dendritische Zellen,

prozessieren die Influenza-Proteine zu Peptiden, die auf der Zelloberfläche von Major-Histocompatibility-Complex(MHC)-Molekülen präsentiert werden (Abb. 2*(c)*). Dendritische Zellen wandern von der Injektionsstelle zu den lokalen Lymphknoten und durchlaufen dabei einen Reifungsprozess (Abb. 2*(d)*). Mit zunehmendem Alter verringern sich die Fähigkeiten zur **Phagozytose**

Phagozytose

und zur **Migration**

Migration,

es kommt zu Veränderungen in der Zytokinproduktion, zu einer geringeren Lokalreaktion und schlechterer Antigenprozessierung [4].

Im Lymphknoten erkennen **naive CD4**^**+**^**-T-Helferzellen**

naive CD4^+^-T-Helferzellen

und **zytotoxische CD8**^**+**^**-T-Zellen**

zytotoxische CD8^+^-T-Zellen,

die spezifisch für die Impfantigene sind, diese auf der Oberfläche der DZ (Abb. 2*(e)*). Die Aktivierung naiver T‑Zellen führt zu massiver Proliferation und zur Differenzierung in Effektor- und Gedächtnis-T-Zellen (Abb. 2*(f)*). Wie oben erwähnt stehen im Alter weniger naive T‑Zellen zur Verfügung, und auch die Interaktion zwischen DZ und T‑Zellen ist weniger effizient [5].

Antigene gelangen auch direkt in die Lymphknoten; dort werden sie von **naiven B‑Zellen**

naiven B‑Zellen

erkannt (Abb. 2*(g)*). Auch die Zahl der naiven B‑Zellen ist im Alter reduziert. Nach der Aktivierung proliferieren B‑Zellen und differenzieren zu **antikörperproduzierenden Zellen**

antikörperproduzierenden Zellen

und **Gedächtnis-B-Zellen**

Gedächtnis-B-Zellen.

Diese erste Welle der B‑Zell-Aktivierung führt einige Tage nach Beginn der Immunantwort zur Produktion von IgM-Antikörpern (Abb. 2*(h)*). Aktivierte B‑ und CD4^+^-T-Zellen bilden gemeinsam Keimzentren, in denen die weiteren Schritte der adaptiven Immunantwort ablaufen. Die B‑Zellen durchlaufen einen Klassenwechsel, wodurch statt IgM andere Antikörperklassen (z. B. IgG oder IgA) gebildet werden. Außerdem kommt es zu Punktmutationen in den variablen, antigenbindenden Regionen der Antikörper und einer Selektion von Antikörpern mit hoher Affinität. Für diese Optimierung der Antikörperantwort ist die T‑Zell-Hilfe essenziell (Abb. 2*(i)*). Es werden Gedächtnis-B-Zellen gebildet, und **langlebige Plasmazellen**

langlebige Plasmazellen

wandern ins Knochenmark; dort produzieren sie über Jahre oder sogar Jahrzehnte Antikörper, die im Serum nachweisbar sind (Abb. 2*(j)*). Im Alter sind diese Prozesse aufgrund funktioneller Defizite der B‑Zellen und reduzierter T‑Zell-Hilfe weniger effizient, was mit verringerten Antikörperantworten bei älteren Erwachsenen einhergeht [6].

Das immunologische Gedächtnis ist dafür verantwortlich, dass manche Infektionen nur einmal durchgemacht werden oder bei wiederholtem Kontakt weniger schwer verlaufen. Allerdings ist dies aufgrund der Variabilität vieler Pathogene (z. B. verschiedene Virusstämme oder Serotypen) und der Tatsache, dass nicht gegen alle Pathogene ein ausreichend langlebiges immunologisches Gedächtnis ausgebildet wird, nicht für alle Erkrankungen der Fall. Der durch Impfstoffe induzierte Schutz vor Krankheiten basiert auf Gedächtnis-T- und Gedächtnis-B-Zellen sowie **zirkulierenden Antikörpern**

zirkulierenden Antikörpern.

Der erste Kontakt mit einem Antigen im Kontext einer Impfung oder Infektion führt zu einer **primären Immunantwort**

primären Immunantwort,

die die Bildung von Gedächtniszellen und optimierten Antikörpern auslöst. Bei einer erneuten Exposition proliferieren Gedächtniszellen schnell und differenzieren zu **Effektorzellen**

**Abb. 3:**
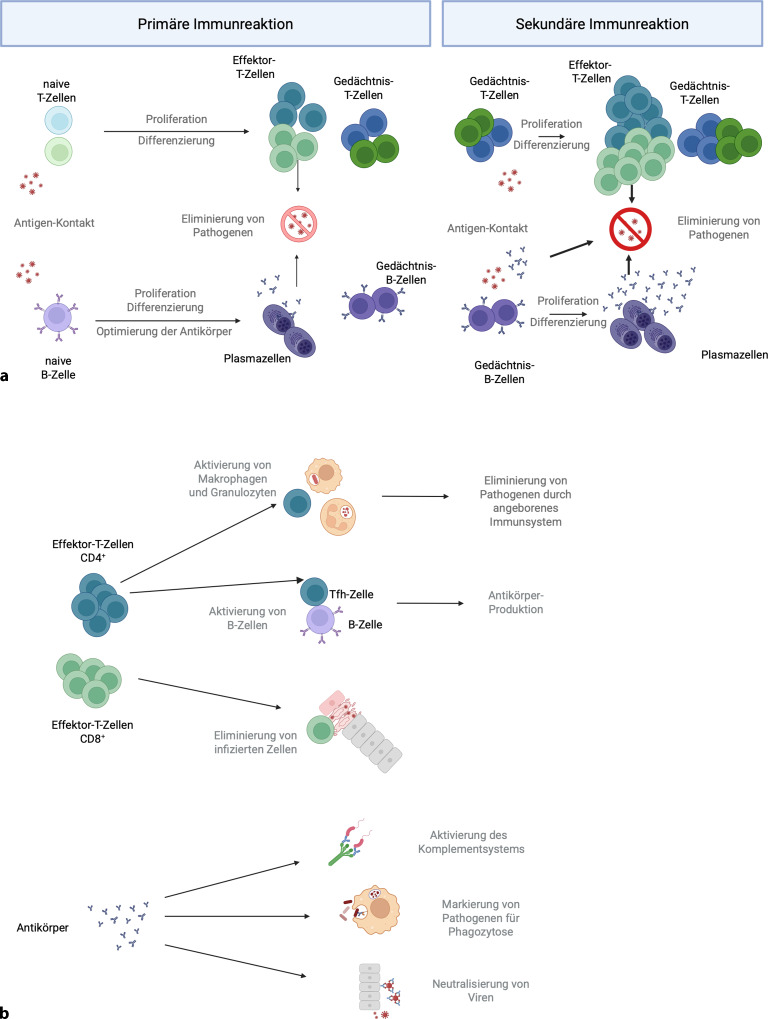
**a** Schematische Darstellung einer primären und sekundären Immunantwort. **b** Schematische Darstellung von Effektorfunktionen für CD4^+^-T-Zellen, CD8^+^-T-Zellen und Antikörper. *Tfh* follikuläre T-Helferzelle. Created in BioRender. Weinberger, B. (2025) https://BioRender.com/kuhvf2c

Effektorzellen,

was eine wesentlich schnellere und effektive Immunantwort im Vergleich zur Primärantwort bedeutet (Abb. 3a). Effektor-CD4^+^-T-Zellen verstärken die Funktionen des angeborenen Immunsystems, z. B., indem sie Makrophagen und andere antigenpräsentierende Zellen aktivieren. Darüber hinaus laufen **sekundäre Immunantworten**

sekundäre Immunantwort

in den Keimzentren, in denen T‑Zellen und B‑Zellen interagieren, effektiver ab, was die Antikörperproduktion verbessert. Effektor-CD8^+^-T-Zellen erkennen infizierte Zellen und eliminieren diese, um die Ausbreitung des Pathogens zu verhindern. Antikörper neutralisieren Pathogene, indem sie z. B. den Eintritt von Viren in Zielzellen blockieren. Sie verstärken außerdem die angeborenen Immunantworten, da Zellen des angeborenen Immunsystems und das **Komplementsystem**

Komplementsystem

Pathogene, die Antikörper gebunden haben, erkennen und bekämpfen (Abb. 3b). Funktionelle Defizite der Immunzellen, z. B. in den Bereichen Signaltransduktion, Metabolismus und Zytokinproduktion, können zu reduzierten Effektorfunktionen und einer verringerten Pathogenabwehr führen [7, 8].

#### Merke

Im Alter kommt es zu charakteristischen Veränderungen, die alle Bestandteile des Immunsystems betreffen.

## Klinische Implikationen

### Chronische Entzündungen und Inflammaging

Akute Immunreaktionen, z. B. bei der Abwehr von Pathogenen oder Gewebereparatur, führen zu transienten Entzündungsreaktionen. Im Alter treten jedoch schwache, aber langanhaltende Entzündungen auf, die altersbedingte Krankheiten fördern.

Aktivierte Zellen des angeborenen Immunsystems, die aufgrund altersbedingter Defekte Pathogene nicht vollständig eliminieren, und hochdifferenzierte Effektorzellen des adaptiven Immunsystems produzieren proinflammatorische Zytokine und treiben chronische Entzündungen voran. Seneszente Zellen, Zellschäden, mitochondriale Dysfunktion und Veränderungen im Darmmikrobiom begünstigen diesen Zustand, der auch als *Inflammaging* bezeichnet wird, ebenfalls [9]. Entzündungen tragen zum Alterungsprozess selbst bei und begünstigen viele altersassoziierte Erkrankungen [10].

Sie spielen eine bedeutende Rolle bei der Entstehung und Progression altersbedingter kardiovaskulärer Erkrankungen und können sowohl lokal im Herzen und in den Gefäßen als auch systemisch vorkommen [11]. Während in der Vergangenheit davon ausgegangen wurde, dass pathologische Veränderungen **neurodegenerativer Erkrankungen**

neurodegenerativer Erkrankungen,

wie z. B. die Aggregation von Proteinen bei M. Parkinson oder Alzheimer-Krankheit, zu Entzündungsreaktionen führen, wurde in den letzten Jahren gezeigt, dass umgekehrt proinflammatorische Faktoren z. B. des alternden Immunsystems das Auftreten dieser Erkrankungen begünstigen [12].

#### Merke

Chronische Entzündungsreaktionen begünstigen viele altersassoziierte Erkrankungen.

### Krebs

Altersassoziierte Veränderungen des Immunsystems beeinflussen die Entstehung, das Wachstum und die Metastasierung von Tumoren. Proinflammatorische Substanzen fördern **Zellproliferation**

Zellproliferation

und **DNA-Schäden**

DNA-Schäden,

was das Tumorwachstum begünstigt. In vielen Fällen werden Tumorzellen immunologisch erkannt und eliminiert. Die reduzierte Zahl von naiven T‑Zellen und ein eingeschränktes Antigenrepertoire beeinträchtigen die Erkennung von Krebsantigenen. Außerdem erkennen und eliminieren zytotoxische T‑Zellen und NK-Zellen im Alter Krebszellen weniger effizient. Tumorzellen können die Immunantwort lokal unterdrücken. Suppressive Zellen wie regulatorische T‑Zellen und myeloide Suppressorzellen, tumorassoziierte Makrophagen und tolerogene DZ schaffen ein **immunsuppressives Milieu**

immunsuppressives Milieu,

das es Tumorzellen ermöglicht, der Immunantwort zu entkommen. Diese Zellen sind teilweise im Alter anders reguliert [13]. Moderne Krebstherapien nutzen immunologische Mechanismen, und der Einfluss altersassoziierter immunologischer Veränderungen auf die Wirksamkeit dieser Therapien wird intensiv untersucht [14].

### Autoimmunität

Bei Autoimmunerkrankungen greift das Immunsystem fälschlicherweise gesunde Zellen des eigenen Körpers an. Die Alterung des Immunsystems trägt zur Entwicklung von Autoimmunität bei. Chronische Entzündungsprozesse, funktionelle Veränderungen von regulatorischen T‑Zellen, die normalerweise Immunantworten steuern und regulieren, und die Produktion von Autoantikörpern durch gealterte B‑Zellen können zu Autoimmunreaktionen beitragen.

Jüngste Studien stützen die These, dass Autoimmunität u. a. auf die **altersbedingte Dysregulation**

altersbedingte Dysregulation

wichtiger zellulärer Prozesse zurückzuführen ist, die zu genomischer Instabilität, abnehmender mitochondrialer Funktion, sowie beeinträchtigter Proteostase, lysosomalem Abbau und Autophagie in Effektor-Zellen führen [15].

### Anfälligkeit für Infektionen

Ältere Erwachsene sind anfälliger für Infektionen, die oft schwerer verlaufen [16]. Gründe sind Immunseneszenz, altersbedingte organspezifische physiologische Veränderungen (z. B. reduzierte Nierenfunktion, Veränderung der Haut- und Schleimhautbarrieren, verringerter Hustenreflex und Zilienbewegung in den Atemwegen etc.), Mangelernährung und chronische Komorbiditäten, aber auch **soziale Gesundheitsfaktoren**

soziale Gesundheitsfaktoren

(z. B. Aufenthalt in einem Pflegeheim, schlechter Zugang zu Pflege). Das zentrale geriatrische Syndrom der Gebrechlichkeit (**Frailty**

Frailty,

[17]) erhöht die Häufigkeit und die Schwere von Infektionen, gemessen z. B. an der Hospitalisierungsrate [18]. Atypische Symptome bei älteren Menschen erschweren die Diagnostik und Behandlung von Infektionen. Ursächlich sind sensorische Einschränkungen, begleitende geriatrische Syndrome wie Demenz, **Polypharmazie**

Polypharmazie

und die **Multimorbidität**

Multimorbidität,

deren Symptomatik klassische Infektionszeichen verändern oder unterdrücken kann.

Neben der akuten Erkrankung bergen Infektionen speziell für ältere Personen zusätzliche Risiken. Bestehende Grunderkrankungen können sich im Zuge einer Infektion verschlechtern, und das Risiko für **kardiovaskuläre Ereignisse**

kardiovaskuläre Ereignisse,

wie Herzinfarkt und Schlaganfall, ist in den Wochen nach einer Infektion deutlich erhöht. Das Risiko für einen Herzinfarkt ist z. B. in der Woche nach einer Influenza-Diagnose ca. 6-mal höher als im Vergleichszeitraum [19]. Oftmals erholen sich ältere Patienten nach einer Infektion nicht mehr vollständig. In einer Studie in den USA wurde gezeigt, dass bei 20 % der hospitalisierten, älteren Influenza-PatientInnen nach der Infektion eine funktionelle Verschlechterung des Allgemeinzustands auftritt [20]. Auch das Risiko eines **Delirs**

Delirs

und/oder einer **Demenz**

Demenz

muss bei geriatrischen PatientInnen im Kontext von Infektionen berücksichtigt werden.

#### Merke

Infektionen bei älteren Menschen haben oft weitreichendere Folgen, die über das Akutereignis hinausgehen.

### Impfungen für ältere Erwachsene

Impfungen sind eine wichtige Präventionsmaßnahme zum Erhalt der Gesundheit und zur Verbesserung der Lebensqualität, insbesondere im Alter. Sie können akute Infektionen, schwere Verläufe und die weitreichenden Folgen von Infektionserkrankungen verhindern. Eine Metaanalyse zeigt, dass die Impfung gegen Influenza das Risiko für einen Herzinfarkt bzw. kardiovaskulären Tod um 30 % bzw. 33 % reduzierten kann [21]. Viele Länder haben spezielle Impfempfehlungen für ältere Erwachsene, darunter Impfungen gegen Influenza, Pneumokokken, Herpes zoster, respiratorisches-Synzytialvirus (RSV) und COVID-19 sowie Auffrischungen für die Impfungen gegen Tetanus, Diphtherie, Pertussis und Frühsommer-Meningoenzephalitis (FSME, je nach Region). Aktuelle Empfehlungen finden sich in den **nationalen Impfplänen**

nationalen Impfplänen,

die regelmäßig aktualisiert werden.

- **Deutschland: **https://www.rki.de/DE/Themen/Infektionskrankheiten/Impfen/Staendige-Impfkommission/Empfehlungen-der-STIKO/Empfehlungen/Impfkalender.html
- **Österreich: **https://www.sozialministerium.gv.at/Themen/Gesundheit/Impfen/Impfplan-Oesterreich.html
- **Schweiz: **https://www.bag.admin.ch/de/schweizerischer-impfplan

Viele Impfstoffe sind bei älteren Erwachsenen weniger immunogen und wirksam als bei jüngeren Personen. Neben dem Alter können auch chronische Erkrankungen (z. B. Diabetes mellitus, chronisch obstruktive Lungenerkrankung [COPD], Nieren- oder Herzinsuffizienz) die Immunogenität und Wirksamkeit von Impfungen verringern.

Die Entwicklung optimierter und neuer Impfstoffe für die ältere Bevölkerung hat in den letzten Jahren und Jahrzehnten große Fortschritte gebracht [22]. Eine Strategie, die Immunantwort bei älteren Personen zu verbessern, ist der Einsatz von Impfstoffen mit höherer Antigendosis. Ein **Hochdosis-Influenza-Impfstoff**

Hochdosis-Influenza-Impfstoff

(60 µg Hämagglutinin/Stamm statt 15 µg) induziert höhere Antikörperkonzentrationen und zeigt sowohl in randomisierten Studien [23] als auch in retrospektiven Kohortenstudien [24] eine verbesserte Wirksamkeit bei älteren Menschen. Gegen Influenza steht außerdem ein **adjuvantierter Impfstoff**

adjuvantierter Impfstoff

zur Verfügung, der neben den Virusantigenen das Adjuvans MF59, eine squalenhaltige Öl-in-Wasser-Emulsion, enthält. Adjuvanzien verstärken die angeborene Immunantwort an der Injektionsstelle und verbessern die adaptive Immunantwort. Auch dieser Impfstoff zeigt eine verbesserte Wirksamkeit im Vergleich mit dem Standardimpfstoff [25]. Viele Länder empfehlen für ältere Erwachsene explizit den präferenziellen Einsatz dieser Impfstoffe gegenüber dem Standardimpfstoff.

Auch bei der Impfung gegen **Herpes zoster**

Herpes zoster

hat sich der Einsatz eines Adjuvans bewährt. Die Primärinfektion mit **Varicella-zoster-Virus**

Varicella-zoster-Virus

führt zum klinischen Bild der Varizellen (Windpocken) und findet meist in der Kindheit statt. In den Neuronen der sensorischen Ganglien etabliert sich eine lebenslange, virale Latenz. Ein attenuierter Lebendimpfstoff für Kinder verhindert die Primärinfektion sehr effizient. Bei infizierten Personen kommt es im Laufe des Lebens immer wieder zu **viralen Reaktivierungen**

viralen Reaktivierungen,

die meist immunologisch kontrolliert werden und deshalb asymptomatisch verlaufen. Ist die immunologische Kontrolle jedoch nicht ausreichend, z. B. aufgrund von Immunsuppression oder eines altersbedingten Absinkens der spezifischen Immunantwort, erfolgt eine symptomatische Reaktivierung mit der Auslösung eines Herpes zoster (Gürtelrose). Die Auftretenshäufigkeit von Herpes zoster steigt ab dem ca. 50. Lebensjahr deutlich an [26], und auch Komplikationen, wie z. B. die **Post-Zoster-Neuralgie**

Post-Zoster-Neuralgie

(PZN), die oft mit gravierenden Alltagseinschränkungen einhergeht, sind im Alter häufiger. Der erste Erwachsenen-Impfstoff gegen Herpes zoster enthielt eine höhere Dosis des gleichen attenuierten Virusstamms, der in der Varizellen-Impfung für Kinder eingesetzt wird. Die Wirksamkeit gegen Herpes zoster betrug jedoch nur ca. 50 % und war bei Personen über 70 Jahren nochmals niedriger [27]. Seit 2018 ist in Europa ein adjuvantierter Proteinimpfstoff gegen Herpes zoster zugelassen. Dieser Impfstoff enthält das rekombinant hergestellte virale Oberflächenprotein gE und ist mit AS01_B_ adjuvantiert, einer in Liposomen formulierten Kombination aus Monophosphoryllipid (MPL) A und dem Saponin QS-21. Diese Impfung zeichnet sich durch eine sehr hohe Wirksamkeit über 90 % sogar in der Altersgruppe > 80 Jahren aus [28], die für mindestens 10 Jahre auf hohem Niveau bleibt [29]. Dieser Impfstoff kann auch bei Personen unter Immunsuppression, die ein hohes Risiko für einen Herpes zoster haben, eingesetzt werden.

Das Adjuvans AS01_B_ ist auch in einem der vor Kurzem zugelassenen Impfstoffe gegen das RSV enthalten. Neben diesem adjuvantierten Proteinimpfstoff wurde nahezu zeitgleich ein weiterer Proteinimpfstoff ohne Adjuvans zugelassen. Beide Präparate enthalten das virale Fusionsprotein F. Außerdem kam kurze Zeit später ein mRNA-Impfstoff auf den Markt, der für das F‑Protein codierende mRNA in Lipidnanopartikeln enthält. Die 3 Impfstoffe zeigten in den Zulassungsstudien eine hohe Wirksamkeit gegen Infektionen der unteren Atemwege [30, 31, 32], die sich nach der Einführung auch in Anwendungsbeobachtungen bestätigte [33]. Die Wirksamkeit ist derzeit für 2 bis 3 Jahre gezeigt. Mit Empfehlungen für Wiederholungsimpfungen ist in naher Zukunft zu rechnen.

Die **mRNA-Impfstoffe**

mRNA-Impfstoffe

wurden erstmals gegen das Severe Acute Respiratory Syndrome Coronavirus 2 (SARS-CoV-2) eingesetzt. Dabei fanden sich auch für ältere Erwachsene eine gute Immunogenität [34] und klinische Wirksamkeit gegen schwere Erkrankungen [35]. Inzwischen wurde gezeigt, dass neutralisierende Antikörper neue Virusvarianten schlechter erkennen, und es wurden gezielt Impfstoffe gegen neue Varianten entwickelt. Die Wirksamkeit gegen symptomatische Erkrankungen ist von relativ kurzer Dauer, der Schutz vor schweren Erkrankungen, Hospitalisierungen und Tod ist jedoch hoch. Zwei Studien aus den USA schätzen, dass eine Auffrischungsimpfung gegen COVID-19 in der Saison 2024–2025 COVID-assoziierte Hospitalisierungen in Personen über 65 Jahren im Vergleich zu Personen ohne Auffrischung in diesem Jahr um fast die Hälfte senkt (45 %, 95%-Konfidenzintervall [95%-KI]:36–53 und 46 %, 95%-KI:26–60; [36]). Diese Daten bestätigen die Empfehlungen für regelmäßige Auffrischungsimpfungen.

Gegen **Streptococcus pneumoniae**

*Streptococcus pneumoniae*

(Pneumokokken) standen in der Vergangenheit verschiedene Impfstoffe zur Verfügung. Es gibt ca. 100 Serotypen von Pneumokokken, die sich anhand ihrer **Polysaccharidkapseln**

Polysaccharidkapseln

unterscheiden. Die Immunantwort gegen die Polysaccharide ist spezifisch für die einzelnen Serotypen. Die Auswahl der in einem Impfstoff enthaltenen Serotypen ist also von entscheidender Bedeutung. Der erste Pneumokokken-Impfstoff war ein 23-valenter Polysaccharid-Impfstoff. Reine Polysaccharid-Impfstoffe lösen eine IgM-lastige, relativ kurzlebige Antikörperantwort aus und sind in Kleinkindern nicht immunogen. Für die Impfung von Säuglingen wurde zunächst ein 7‑valenter Konjugatimpfstoff entwickelt, der die bakteriellen Polysaccharide an ein Trägerprotein (Diphtherie-Toxoid) koppelt. Dieser Impfstoff ist auch für Säuglinge geeignet und ermöglicht eine verbesserte Antikörperantwort [37]. Die Zahl der Serotypen in **Konjugatimpfstoffen**

**Abb. 4:**
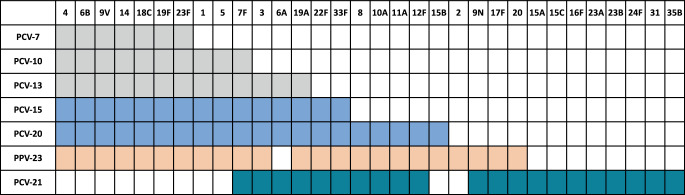
Serotypen in Polysaccharid(PPV)- und Konjugat(PCV)-Impfstoffen gegen *S.* *pneumoniae*. *PPV* „pneumococcal polysaccharide vaccine“, *PCV* „pneumococcal conjugate vaccine“

Konjugatimpfstoffen

wurde laufend erhöht auf derzeit 15 bzw. 20, wobei sich die Zusammensetzung dem 23-valenten Polysaccharid-Impfstoff annäherte. Hohe Durchimpfungsraten bei Kindern sorgen dafür, dass die Zirkulation der in diesen Impfstoffen enthaltenen Serotypen auch bei Erwachsenen zurückgeht. Im Frühjahr 2025 wurde in Europa ein weiterer Konjugatimpfstoff gegen Pneumokokken zugelassen. Dieser Impfstoff enthält 21 Serotypen, unterscheidet sich jedoch in der Auswahl der Serotypen deutlich von den anderen Impfstoffen und deckt speziell die Serotypen ab, die bei älteren Erwachsenen für schwere Infektionen verantwortlich sind ([38]; Abb. 4). In Österreich wird die Verwendung dieses Impfstoffs für ältere Erwachsene empfohlen.

Auch verkürzte Intervalle von **Auffrischungsimpfungen**

Auffrischungsimpfungen

für ältere Personen (Tetanus, Diphtherie, Pertussis, FSME) tragen zu einem verbesserten Schutz dieser Altersgruppe bei, da dem Absinken der Antikörperkonzentrationen unter einen schützenden Wert entgegengewirkt werden kann.

#### Merke

In den letzten Jahren wurden große Fortschritte bei der Verbesserung bestehender und der Entwicklung neuer Impfstoffe für ältere Erwachsene erzielt.

Ein entscheidender Punkt ist jedoch die Umsetzung von Impfungen in die routinemäßige medizinische Versorgung von Erwachsenen. Die **Durchimpfungsraten**

Durchimpfungsraten

bei älteren Erwachsenen liegen deutlich unter den angestrebten Zielen.

Detaillierte Überlegungen zur Erhöhung der Durchimpfungsraten sprengen den Rahmen dieses Artikels, einige entscheidende Punkte sollen jedoch erwähnt werden. Der erste Schritt ist die Schaffung möglichst vieler Gelegenheiten zum Impfen. Ärzte im niedergelassenen Bereich (Allgemeinmedizin und verschiedene Fachrichtungen) sind mit der Impfung von Erwachsenen betraut. Eine Ausdehnung der Zuständigkeiten z. B. auf stationäre Einrichtungen, Apotheken oder Impfzentren könnte zusätzliche Angebote schaffen. Die Dokumentation von Impfungen ist essenziell für individuelle Entscheidungen und Erinnerungen. **Impfskepsis**

Impfskepsis

ist ein wachsendes Problem, dem mit viel individueller Aufklärung und Überzeugungsarbeit entgegengewirkt werden kann. Entscheidend sind zum einen eine umfassende Kenntnis der Datenlage, zum anderen aber auch eine effektive, personalisierte Kommunikationsstrategie. Wichtig ist, das Risiko der Erkrankung und möglicher Folgeerscheinungen realistisch einzuschätzen, auf Bedenken wegen Nebenwirkungen adäquat einzugehen, diese wissenschaftlich fundiert zu entkräften und die Schutzwirkung der Impfung entsprechend darzustellen. Die persönliche Einstellung des Arztes/der Ärztin ist entscheidend. Umso wichtiger ist es, dass auch das Personal im Gesundheitswesen selbst geimpft ist. Logistische Hürden, wie z. B. zusätzliche Wegstrecken, um den Impfstoff in der Apotheke abzuholen, müssen abgebaut werden. Ein unkomplizierter, niederschwelliger Zugang zu Impfungen ist, neben der finanziellen Abdeckung, essenziell, um die Durchimpfungsraten zu steigern.

Impfungen haben das Potenzial, Leben zu retten, die Gesundheit älterer Menschen zu verbessern und Kosten im Gesundheitswesen zu senken. Immunologische Forschung, Impfstoffentwicklung, Impfprogramme und **Informationskampagnen**

Informationskampagnen

müssen weiter vorangetrieben werden, um die wachsende ältere Bevölkerung optimal zu schützen.

#### Merke

Eine Steigerung der Durchimpfungsraten in der älteren Bevölkerung ist dringend notwendig.

### Strategien zur Bekämpfung der Immunalterung

Interventionen zur Verzögerung oder zur Umkehrung der Immunseneszenz könnten altersassoziierte Krankheiten positiv beeinflussen. Ein **gesunder Lebensstil**

gesunder Lebensstil

(Ernährung, Bewegung, Schlaf, Stressreduktion) stärkt das Immunsystem und wirkt chronischen Entzündungen entgegen. Impfungen verhindern schwere Infektionen und deren Folgen. Gezielte Ansätze zur „Verjüngung“ des Immunsystems werden erforscht, befinden sich aber oft noch in frühen Entwicklungsstadien. Kalorienrestriktion und Fasten zeigen in Studien positive Effekte auf Alterung und Immunsystem, erfordern jedoch eine langfristige Umsetzung. Antiinflammatorische und antioxidative Substanzen sowie **Senolytika**

Senolytika,

die seneszente Zellen eliminieren, haben in Tiermodellen vielversprechende Ergebnisse gezeigt, sind jedoch für den Einsatz bei Menschen noch nicht ausreichend erforscht. Ansätze, die Proliferationsfähigkeit seneszenter Zellen wiederherzustellen oder z. B. den Thymus zu regenerieren, klingen verheißungsvoll, sind jedoch potenziell mit Risiken wie z. B. Tumorentstehung verbunden und noch nicht ausreichend getestet.

## Fazit für die Praxis

- Im Alter kommt es zu komplexen Veränderungen des angeborenen und adaptiven Immunsystems. Dieses Phänomen ist als Immunseneszenz bekannt.
- Immunseneszenz und chronische Entzündungsreaktionen (Inflammaging) begünstigen viele altersassoziierte Erkrankungen.
- Impfungen sind v. a. im Alter eine wichtige Präventionsmaßnahme, die nicht nur akute Infektionen, sondern auch deren weitreichende Folgen verhindern können.
- Die Entwicklung optimierter und neuer Impfstoffe für die ältere Bevölkerung hat in den letzten Jahren und Jahrzehnten große Fortschritte gebracht.
- Die Durchimpfungsraten bei älteren Erwachsenen liegen für alle Impfungen deutlich unter den angestrebten Zielen. Eine Steigerung der Durchimpfungsraten in der älteren Bevölkerung ist dringend notwendig.

## CME-Fragebogen

Die Entwicklung welcher immunologisch relevanten Zellart wird durch Involution des Thymus besonders eingeschränkt?

B‑Zellen

Dendritische Zellen

Makrophagen

Mastzellen

T‑Zellen

Welche Veränderung bei der Hämatopoese ist im Alter zu beobachten?

Die Hämatopoese verlagert sich vom Knochenmark in die Milz.

Es werden weniger myeloide Vorläuferzellen gebildet.

Die Fähigkeit zur Selbsterneuerung der hämatopoetischen Stammzellen nimmt zu.

Es werden weniger lymphoide Vorläuferzellen gebildet.

Es werden keine natürlichen Killerzellen (NK-Zellen) mehr gebildet.

Welche Veränderung des angeborenen Immunsystems ist im Alter zu beobachten?

Die Migrationsfähigkeit der Makrophagen nimmt zu.

Die Fähigkeit der Makrophagen zur Phagozytose nimmt ab.

Die Zahl der naiven B‑Zellen nimmt ab.

Die Fähigkeit der Makrophagen, Antikörper zu produzieren, nimmt ab.

Die Zellen das angeborenen Immunsystems stellen die Zytokinproduktion ein.

Welche Veränderung von T‑Zellen ist im Alter zu beobachten?

Die Zahl der naiven T‑Zellen nimmt ab .

Die Zahl der Effektor-T-Zellen nimmt ab.

Es werden nur noch CD4^+^-T-Zellen gebildet.

Die CD8^+^-T-Zellen können virusinfizierte Zellen besonders effizient eliminieren.

T‑Zellen können nicht mehr proliferieren.

Welche Impfung ist für ältere Erwachsene *nicht* relevant?

Haemophilus influenzae

Herpes zoster

Influenza

Respiratorisches Synzytialvirus (RSV)

COVID-19

Welche dieser Anpassungen wird bei Influenza-Impfstoffen angewandt, um sie für ältere Erwachsene wirksamer zu machen?

Impfstoff mit Aluminiumsalzen als Adjuvans

Impfstoff mit höherer Antigendosis

Intravenöse Gabe des Impfstoffs

Impfstoff mit 10 unterschiedlichen Influenza-Stämmen

Gabe von 3 Impfdosen im Abstand von jeweils 2 Wochen

Was ist die Besonderheit des 2025 zugelassenen 21-valenten Konjugatimpfstoffs gegen Pneumokokken?

Er deckt speziell die Serotypen, die bei älteren Erwachsenen für schwere Infektionen verantwortlich sind, ab.

Er enthält als einziger der Pneumokokken-Impfstoffe ein Adjuvans.

Er wirkt nicht nur gegen Pneumokokken, sondern auch andere Erreger von Pneumonien.

Er sollte nur im Frühling verabreicht werden.

Er enthält nicht nur konjugierte Polysaccharide, sondern auch 3 Pneumokokkenproteine.

Der durch Impfstoffe induzierte Schutz vor Krankheiten basiert auf:

Verstärkter Funktion der Granulozyten

Bildung von Gedächtnis-T- und Gedächtnis-B-Zellen und der Produktion von Antikörpern

Erhöhten Werten des C‑reaktiven Proteins (CRP)

Verstärkter Seneszenz der Immunzellen

Verbesserter Hämatopoese

Inflammaging bewirkt ein erhöhtes Risiko für altersbedingte Krankheiten wie Herz-Kreislauf-Erkrankungen, Diabetes, Alzheimer-Krankheit und Krebs. Es beschleunigt den Alterungsprozess und führt zu Funktionseinschränkungen der Organe und Gewebe. *Nichts* damit zu tut hat/haben …

die Bildung proinflammatorischer Zytokine bei unvollständiger Eliminierung von Pathogenen.

die Produktion proinflammatorischer Substanzen durch seneszente Zellen.

altersassoziierte Sehschäden,

die Dysfunktion der Mitochondrien.

Veränderungen im Darmmikrobiom.

Welcher dieser Impfstoffe enthält ein Adjuvans?

15-valenter Konjugatimpfstoff gegen Pneumokokken

Rekombinanter Impfstoff gegen Herpes zoster

Attenuierter Lebendimpfstoff gegen Masern

mRNA-Impfstoff gegen das respiratorische Synzytialvirus (RSV)

Hochdosisimpfstoff gegen Influenza

## References

[1] Walford RL (1964) The immunnological theory of aging. Gerontologist 4:195–197 14289265 10.1093/geront/4.4.195

[2] Li X, Wang J, Hu L, Cheng T (2025) How age affects human hematopoietic stem and progenitor cells and the strategies to mitigate aging. Exp Hematol. 10.1016/j.exphem.2025.104711 39788412 10.1016/j.exphem.2025.104711

[3] Thomas R, Wang W, Su DM (2020) Contributions of age-related thymic involution to immunosenescence and inflammaging. Immun Ageing. 10.1186/s12979-020-0173-8 31988649 10.1186/s12979-020-0173-8 PMC6971920

[4] Montgomery RR, Shaw AC (2015) Paradoxical changes in innate immunity in aging: recent progress and new directions. J Leukoc Biol. 10.1189/jlb.5mr0315-104r 26188078 10.1189/jlb.5MR0315-104R PMC4661037

[5] Pangrazzi L, Weinberger B (2020) T cells, aging and senescence. Exp Gerontol 134:110887. 10.1016/j.exger.2020.110887 32092501 10.1016/j.exger.2020.110887

[6] Frasca D, DIaz A, Romero M et al (2020) B cell immunosenescence. Annu Rev Cell Dev Biol. 10.1146/annurev-cellbio-011620-034148 33021823 10.1146/annurev-cellbio-011620-034148 PMC8060858

[7] Doherty TM, Weinberger B, Didierlaurent A, Lambert PH (2025) Age-related changes in the immune system and challenges for the development of age-specific vaccines. Ann Med 57:2477300. 10.1080/07853890.2025.2477300/ASSET/38C31E64-33DC-4697-AFB1-6D0C9B4B276C/ASSETS/GRAPHIC/IANN_A_2477300_F0003_C.JPG 40110678 10.1080/07853890.2025.2477300 PMC11926906

[8] Liu Z, Liang Q, Ren Y et al (2023) Immunosenescence: molecular mechanisms and diseases. Signal Transduct Target Ther. 10.1038/s41392-023-01451-2 37179335 10.1038/s41392-023-01451-2 PMC10182360

[9] Franceschi C, Bonafè M, Valensin S et al (2006) Inflamm-aging: an evolutionary perspective on immunosenescence. Ann N Y Acad Sci 908:244–254. 10.1111/j.1749-6632.2000.tb06651.x 10.1111/j.1749-6632.2000.tb06651.x 10911963

[10] Franceschi C, Campisi J (2014) Chronic inflammation (inflammaging) and its potential contribution to age-associated diseases. J Gerontol A Biol Sci Med Sci. 10.1093/gerona/glu057 24833586 10.1093/gerona/glu057

[11] Ajoolabady A, Pratico D, Vinciguerra M et al (2023) Inflammaging: mechanisms and role in the cardiac and vasculature. Trends Endocrinol Metab. 10.1016/j.tem.2023.03.005 37076375 10.1016/j.tem.2023.03.005

[12] Zhang W, Xiao D, Mao Q, Xia H (2023) Role of neuroinflammation in neurodegeneration development. Signal Transduct Target Ther. 10.1038/s41392-023-01486-5 37433768 10.1038/s41392-023-01486-5 PMC10336149

[13] Rocamora-Reverte L, Melzer FL, Würzner R, Weinberger B (2021) The complex role of regulatory T cells in immunity and aging. Front Immunol 11:616949. 10.3389/fimmu.2020.616949 33584708 10.3389/fimmu.2020.616949 PMC7873351

[14] Poropatich K, Fontanarosa J, Samant S et al (2017) Cancer immunotherapies: are they as effective in the elderly? Drugs Aging. 10.1007/s40266-017-0479-1 28707274 10.1007/s40266-017-0479-1

[15] Zheng Y, Liu Q, Goronzy JJ, Weyand CM (2023) Immune aging—a mechanism in autoimmune disease. Semin Immunol. 10.1016/j.smim.2023.101814 37542986 10.1016/j.smim.2023.101814 PMC10663095

[16] Gavazzi G, Krause K (2002) Ageing and infection. Lancet 2:659–666 10.1016/s1473-3099(02)00437-1 12409046

[17] Clegg A, Young J, Iliffe S et al (2013) Frailty in elderly people. Lancet 381:752–762. 10.1016/S0140-6736(12)62167-9 23395245 10.1016/S0140-6736(12)62167-9 PMC4098658

[18] Iwai-Saito K, Shobugawa Y, Aida J, Kondo K (2021) Frailty is associated with susceptibility and severity of pneumonia in older adults (A JAGES multilevel cross-sectional study). Sci Rep. 10.1038/s41598-021-86854-3 33846416 10.1038/s41598-021-86854-3 PMC8041848

[19] Kwong JC, Schwartz KL, Campitelli MA et al (2018) Acute myocardial infarction after laboratory-confirmed influenza infection. N Engl J Med 378:345–353. 10.1056/nejmoa1702090 29365305 10.1056/NEJMoa1702090

[20] Andrew MK, MacDonald S, Godin J et al (2021) Persistent functional decline following hospitalization with influenza or acute respiratory illness. J Am Geriatr Soc. 10.1111/jgs.16950 33294986 10.1111/jgs.16950 PMC7984066

[21] Omidi F, Zangiabadian M, Shahidi Bonjar AH et al (2023) Influenza vaccination and major cardiovascular risk: a systematic review and meta-analysis of clinical trials studies. Sci Rep. 10.1038/s41598-023-47690-9 37981651 10.1038/s41598-023-47690-9 PMC10658159

[22] Pangrazzi L, Weinberger B (2025) Recent developments of vaccines for older adults: adjuvants and beyond. Hum Vaccin Immunother 21:2517931. 10.1080/21645515.2025.2517931 40540317 10.1080/21645515.2025.2517931 PMC12934143

[23] DiazGranados CA, Dunning AJ, Kimmel M et al (2014) Efficacy of high-dose versus standard-dose influenza vaccine in older adults. N Engl J Med 371:635–645. 10.1056/NEJMoa1315727 25119609 10.1056/NEJMoa1315727

[24] Bricout H, Levant M‑C, Assi N et al (2024) The relative effectiveness of a high-dose quadrivalent influenza vaccine versus standard-dose quadrivalent influenza vaccines in older adults in France: a retrospective cohort study during the 2021–22 influenza season. Clin Microbiol Infect 12:1592–1598. 10.1016/j.cmi.2024.08.012 10.1016/j.cmi.2024.08.012 39187126

[25] Ferdinands JM, Blanton LH, Alyanak E et al (2024) Protection against influenza hospitalizations from enhanced influenza vaccines among older adults: a systematic review and network meta-analysis. J Am Geriatr Soc 72:3875–3889. 10.1111/JGS.19176 39230284 10.1111/jgs.19176 PMC11637296

[26] Kawai K, Gebremeskel BG, Acosta CJ (2014) Systematic review of incidence and complications of herpes zoster: towards a global perspective. BMJ Open 4:e4833. 10.1136/bmjopen-2014-004833 24916088 10.1136/bmjopen-2014-004833 PMC4067812

[27] Oxman MN, Levin MJ, Johnson GR et al (2005) A vaccine to prevent herpes zoster and postherpetic neuralgia in older adults. N Engl J Med 352:2271–2284 15930418 10.1056/NEJMoa051016

[28] Lal H, Cunningham AL, Godeaux O et al (2015) Efficacy of an adjuvanted herpes zoster subunit vaccine in older adults. N Engl J Med 372:2087–2096. 10.1056/NEJMoa1501184 25916341 10.1056/NEJMoa1501184

[29] Strezova A, Diez-Domingo J, Al Shawafi K et al (2022) Long-term protection against herpes zoster by the adjuvanted recombinant zoster vaccine: interim efficacy, Immunogenicity, and safety results up to 10 years after initial vaccination. Open Forum Infect Dis 9:ofac485. 10.1093/ofid/ofac485 36299530 10.1093/ofid/ofac485 PMC9588150

[30] Ison MG, Papi A, Athan E et al (2024) Efficacy and safety of respiratory syncytial virus (RSV) prefusion F protein vaccine (RSVpreF3 OA) in older adults over 2 RSV seasons. Clin Infect Dis 78:1732–1744. 10.1093/CID/CIAE010 38253338 10.1093/cid/ciae010 PMC11175669

[31] Walsh EE, Pérez Marc G, Zareba AM et al (2023) Efficacy and safety of a bivalent RSV prefusion F vaccine in older adults. N Engl J Med. 10.1056/NEJMoa2213836 37018468 10.1056/NEJMoa2213836

[32] Wilson E, Goswami J, Baqui AH et al (2023) Efficacy and safety of an mRNA-based RSV PreF vaccine in older adults. N Engl J Med 389:2233–2244. 10.1056/nejmoa2307079 38091530 10.1056/NEJMoa2307079

[33] Payne AB, Watts JA, Mitchell PK et al (2024) Respiratory syncytial virus (RSV) vaccine effectiveness against RSV-associated hospitalisations and emergency department encounters among adults aged 60 years and older in the USA, October, 2023, to March, 2024: a test-negative design analysis. Lancet 404:1547–1559. 10.1016/S0140-6736(24)01738-0 39426837 10.1016/S0140-6736(24)01738-0 PMC13286031

[34] Walsh EE, Frenck RW, Falsey AR et al (2020) Safety and immunogenicity of two RNA-based Covid-19 vaccine candidates. N Engl J Med 383:2439–2450. 10.1056/nejmoa2027906 33053279 10.1056/NEJMoa2027906 PMC7583697

[35] Haas EJ, Angulo FJ, McLaughlin JM et al (2021) Impact and effectiveness of mRNA BNT162b2 vaccine against SARS-CoV‑2 infections and COVID-19 cases, hospitalisations, and deaths following a nationwide vaccination campaign in Israel: an observational study using national surveillance data. Lancet 397:1819–1829. 10.1016/S0140-6736(21)00947-8 33964222 10.1016/S0140-6736(21)00947-8 PMC8099315

[36] Link-Gelles R, Chickery S, Webber A et al (2025) Interim estimates of 2024–2025 COVID-19 vaccine effectiveness among adults aged ≥ 18 years—VISION and IVY networks, September 2024-January 2025. Mmwr Morb Mortal Wkly Rep 74:73–82. 10.15585/MMWR.MM7406A1 40014628 10.15585/mmwr.mm7406a1 PMC11867580

[37] Pollard AJ, Perrett KP, Beverley PC (2009) Maintaining protection agaisnt invasive bacteria with protein-polysaccharide conjugate vaccines. Nat Rev Immunol 426:422–426 10.1038/nri2494 19214194

[38] Platt HL, Bruno C, Buntinx E et al (2024) Safety, tolerability, and immunogenicity of an adult pneumococcal conjugate vaccine, V116 (STRIDE-3): a randomised, double-blind, active comparator controlled, international phase 3 trial. Lancet Infect Dis 24:1141–1150. 10.1016/S1473-3099(24)00344-X 38964361 10.1016/S1473-3099(24)00344-X

